# Integrated array of coupled exciton–polariton condensates

**DOI:** 10.1515/nanoph-2025-0469

**Published:** 2025-11-19

**Authors:** Pietro Tassan, Etsuki Kobiyama, Jan David Fischbach, Dario Ballarini, Luca Moretti, Lorenzo Dominici, Milena De Giorgi, Daniele Sanvitto, Michael Forster, Ullrich Scherf, Antonis Olziersky, Carsten Rockstuhl, Thomas Jebb Sturges, Rainer F. Mahrt, Darius Urbonas, Thilo Stöferle

**Affiliations:** 54174 IBM Research Europe – Zurich, Rüschlikon, Switzerland; Photonics Laboratory, ETH Zürich, Zürich, Switzerland; Karlsruhe Institute of Technology, Institute of Nanotechnology, Karlsruhe, Germany; CNR Nanotec, Institute of Nanotechnology, Lecce, Italy; Macromolecular Chemistry Group and Wuppertal Center for Smart Materials & Systems (CM@S), Bergische Universität Wuppertal, Wuppertal, Germany; Karlsruhe Institute of Technology, Institute of Theoretical Solid State Physics, Karlsruhe, Germany

**Keywords:** exciton-polaritons, polariton condensate, microcavity, high-contrast grating, cavity array

## Abstract

A central challenge for advancing polariton-based circuits is the controlled and scalable coupling of individual condensates. Existing approaches based on etched or epitaxially grown microcavities are fabrication-intensive and restrict in-plane coupling. To overcome these limitations, we introduce a lithographically defined silicon-based platform of high-contrast grating (HCG) microcavities with a ladder-type π-conjugated polymer. In this system, doublet cavities exhibit mode hybridization into bonding and antibonding states, where coupling is mediated across shared HCG mirrors. Extending the design to arrays, *N*-coupled condensates exhibit systematic red-shifts of the condensate energy, due to delocalization, and a progressive threshold reduction, consistent with extended binding modes. Our experimental results are quantitatively supported by transition-matrix multi-scattering simulations, together with tight-binding modelling. First-order coherence measurements using Michelson interferometry confirm the existence of spatially extended condensates with exponentially decaying temporal coherence. Altogether, these results establish a scalable route toward integrated polariton devices and quantum photonic networks.

## 1 Introduction

Exciton–polaritons, hybrid light–matter quasiparticles that are formed under strong coupling between excitons and photons in optical microcavities [1], have attracted broad interest thanks to their nonlinear response. These systems, when excited at sufficiently high fluences, can undergo exciton-polariton condensation, resulting in the coherent population of a single macroscopically extended state [2], [3]. With organic semiconductors [4], characterized by strongly bound Frenkel excitons, this condensation can take place at room temperature [5], [6], [7], rendering this regime appealing for photonic devices that can operate at ambient conditions [8], [9]. Coupling multiple condensates [10], [11] enables access to Bloch band physics [12] and eventually the simulation of interesting lattice Hamiltonians [13], [14]. Furthermore, it opens the way toward more advanced polariton-based devices [15]. Previous implementations of arrays of polariton condensates have been realized mainly in vertical DBR cavities, using etched lattices [12], [16], [17], [18], [19], [20], [21], and, more recently, using spatial light modulators (SLMs) [22], [23], [24]. In etched arrays, the coupling between lattice sites is achieved by a finite mode overlap between the resonant modes of adjacent sites. Here, the losses through the DBR mirrors, i.e. the outcoupled polariton signal, can only be redirected into neighboring cavities via external optics [15], which inhibits the formation of a common mode due to retardation effects and ultimately limits scalability. In addition to the vertical outcoupling, SLM-based arrays require extensive off-chip free-space optics. Hence, both approaches face fundamental challenges in achieving integrated polaritonic circuitry.

Subwavelength gratings that harness the high refractive index contrast between the grating and the surrounding material have recently emerged as a practical alternative for the realization of mirrors with high, broadband reflectivity [25], suitable also for polariton lasers and condensates in vertical cavities [26] but also in metal [27], [28] and silicon [29] metasurfaces. Planar silicon-based high-contrast gratings (HCGs) enable fully integrated Fabry–Perot-like cavities that are compatible with silicon photonic fabrication, offering highly tunable cavity designs and an extremely compact footprint [30]. Very recently, all-optical polariton transistor action has been demonstrated in such devices [31]. As the cavity axis is parallel to the chip, the photons transmitted through the HCG mirrors can be coupled into nearby HCG cavities, holding promise to enable the realization of larger integrated polaritonic circuits.

Building on our recent work on individual HCG cavities [31], where strong light–matter coupling and polariton condensation were established, here we present arrays of HCG microcavities filled with a conjugated polymer that allow us to create integrated coupled polariton condensates at room temperature. In this architecture, adjacent cavities share HCG mirrors of finite reflectivity, allowing polariton exchange between neighboring sites. This platform combines lithographic scalability with controlled mode hybridization, providing a basis for systematic studies of condensation in such directly coupled, integrated arrays of condensates.

## 2 Results and discussion

### 2.1 Polariton condensation in two coupled HCG cavities

**Figure 1:**
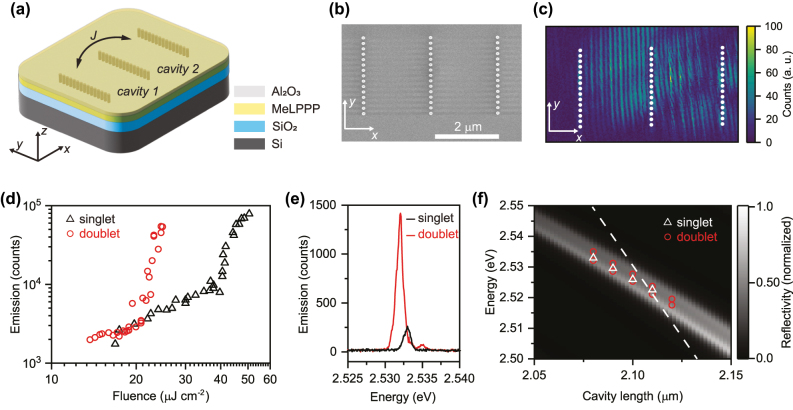
Mode hybridization in HCG cavity doublets. (a) Schematic of a doublet defined by three HCG mirrors forming two contiguously coupled cavities that share the central mirror. *J* denotes the coupling strength between the two resonators. (b) Top-view scanning electron microscopy image of a doublet before deposition of the polymer. (c) Near-field map of a doublet acquired by scattering-type SNOM under 488 nm resonant excitation. The image shows the fourth-harmonic demodulated signal, normalized to its maximum, with the HCG pillar structures overlaid (white dots). Resonant excitation allows direct visualization of the real-space field distribution across the two cavities. (d) Integrated emission counts versus excitation fluence for a single cavity (black triangles) and a doublet (red circles), showing the reduced polariton condensation threshold in the coupled case. (e) Above threshold emission spectra for a singlet (black) and doublet (red), highlighting the energy hybridization in the coupled system. (f) Calculated reflectivity from RCWA as a function of cavity length for cavities in the strong light–matter interaction regime (grey scale map). The experimentally measured energies extracted above threshold are overlaid for singlets (white triangles) and doublets (red circles). As comparison, the dashed line shows the RCWA-calculated resonance energy dependence for a single HCG cavity in the weak coupling regime, using a fixed nondispersive refractive index *n* = 1.86.

The structure (Figure 1a and b) is fabricated on a silicon-on-insulator wafer using established silicon photonics processes, followed by deposition of the ladder-type π-conjugated polymer methyl-ladder poly(para-phenylene) (MeLPPP) and an encapsulation layer (see Methods – Fabrication). In this geometry, the HCG mirrors provide in-plane confinement with a quality factor *Q* of about 300–400 while the polymer acts both as the active medium and as the guiding layer, with vertical confinement provided by total internal reflection [31]. Imaging with scanning near-field optical microscopy (SNOM) under resonant excitation (see Methods – Optical Characterization) reveals the real-space profile of the hybridized cavity mode in a doublet. Here, the measured intensity exhibits nodes and antinodes with a spatial period set by the mode wavelength and effective refractive index. This possibility to image the whole cavity mode is unique to the in-plane HCG geometry and not accessible in conventional vertical DBR cavities (Figure 1c).

The structure is optically excited from the top using pulsed laser illumination (see Methods – Optical Characterization). As the pump fluence increases, the emission shows a light-in-light-out characteristic with a nonlinear rise above threshold, where the threshold fluence differs markedly between a single cavity and a doublet (Figure 1d). Above threshold, a narrow peak emerges in the spectrum of a single cavity, whereas the doublet exhibits a clear splitting into two hybridized modes (Figure 1e) [10], [21], [32]. The splitting arises from the fact that the central HCG mirror has finite reflectivity through which the two resonators are coupled and polaritons can be exchanged between the two cavities. This gives rise to symmetric and antisymmetric modes, similar to hybridized molecular orbitals with a lower-energy bonding state and a higher-energy antibonding state. We study the evolution of their energies by extracting the emission peak positions of singlets and doublets as a function of cavity length and overlaid them on reflectivity maps (Figure 1f) calculated using two-dimensional rigorous coupled wave analysis (RCWA) in the strong exciton-photon coupling regime (see Methods – Photonic Simulations). Detuning the cavity resonance energy with respect to the exciton energy of MeLPPP at 2.71 eV reveals that the measured mode energies follow the simulated lower-polaritonic branch in Figure 1f (RCWA-based strong-coupling model) and are disjunct from the resonance energies expected in the weak-coupling regime, which, together with our prior, more detailed single-device study on the same HCG/MeLPPP platform [31], confirms the strong light–matter interaction regime. The polariton condensation preferentially occurs about 200 meV below the exciton energy due to the vibronically assisted relaxation from the exciton reservoir [15], [33].

### 2.2 Array of integrated polariton condensates

**Figure 2:**
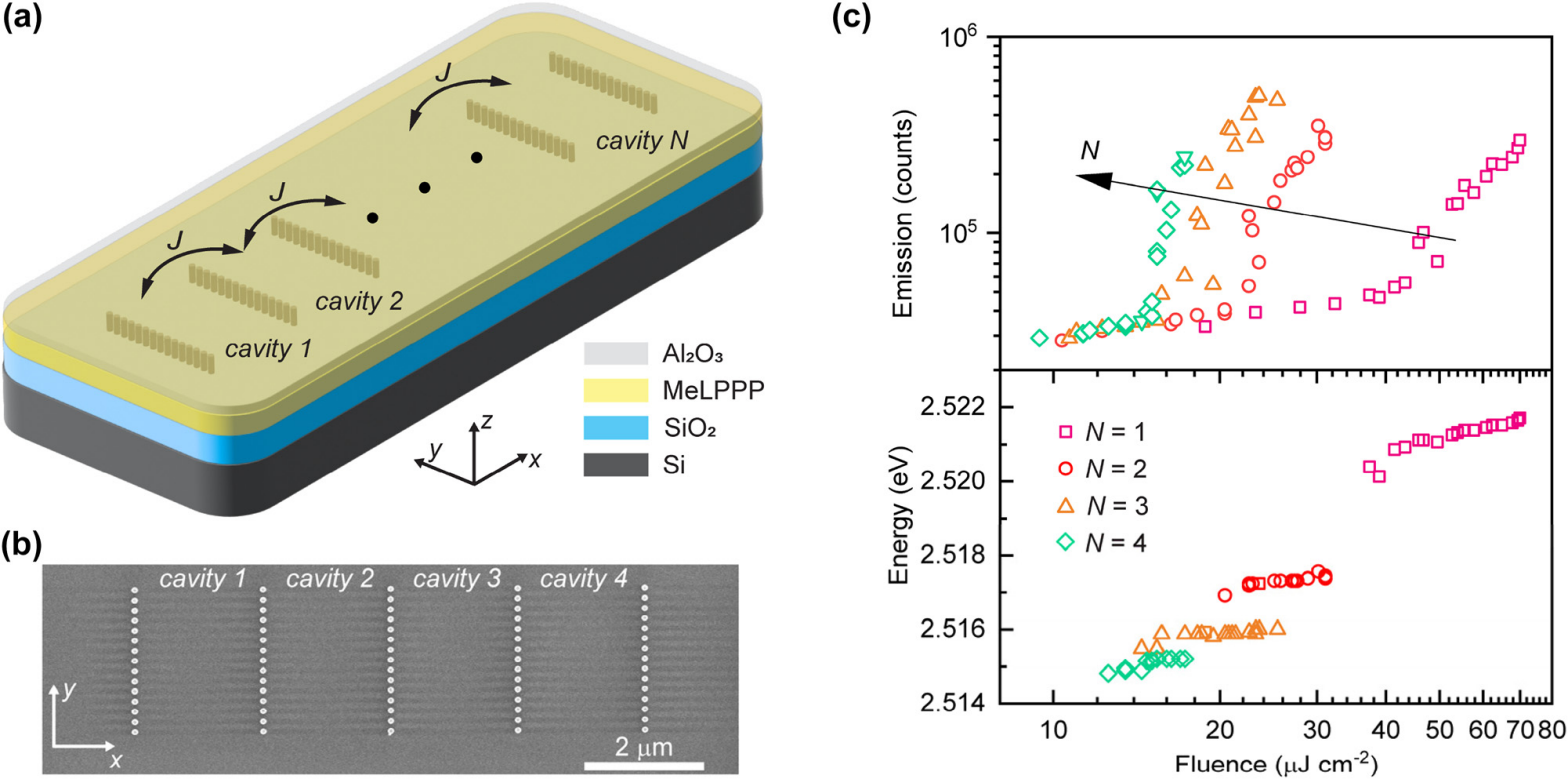
Polariton condensation in arrays of contiguously coupled HCG cavities. (a) Schematic of an array of *N* coupled cavities with shared HCG mirrors. (b) Top-view scanning electron micrograph of a fabricated array for *N* = 4, prior to the polymer deposition. (c, top) Spectrally integrated emission intensity as a function of excitation fluence for arrays with different *N*, showing a progressive reduction of the condensation threshold with increasing cavity number. (c, bottom) Energy of the emission peak as a function of excitation fluence and number of coupled cavities *N*.

Building on this basic doublet, we next extend the concept to larger arrays and study how threshold and condensate energies evolve with the array size. Straightforwardly, this platform allows the realization of linear arrays of *N* contiguously coupled HCG cavities (Figure 2a and b), which we choose to all have the same single-unit cavity length. Our previous studies have shown that despite unavoidable slight fabrication variations, the energetic disorder is sufficiently small allowing to bring them all into resonance with each other, tuned with an accuracy well within the cavity line width [31]. The light-in-light-out curves recorded as a function of excitation fluence show a gradual reduction of the condensation threshold with increasing number of cavities *N* (Figure 2c top panel). The condensate energy in each array exhibits a slight blueshift with increasing pump fluence, typically attributed mainly to saturation of the transition [34]. Increasing the number of coupled cavities *N* induces a systematic red-shift of the condensate energy, consistent with the formation of a delocalized mode that extends across the whole array (Figure 2c bottom panel) [12], [16], [21].

**Figure 3:**
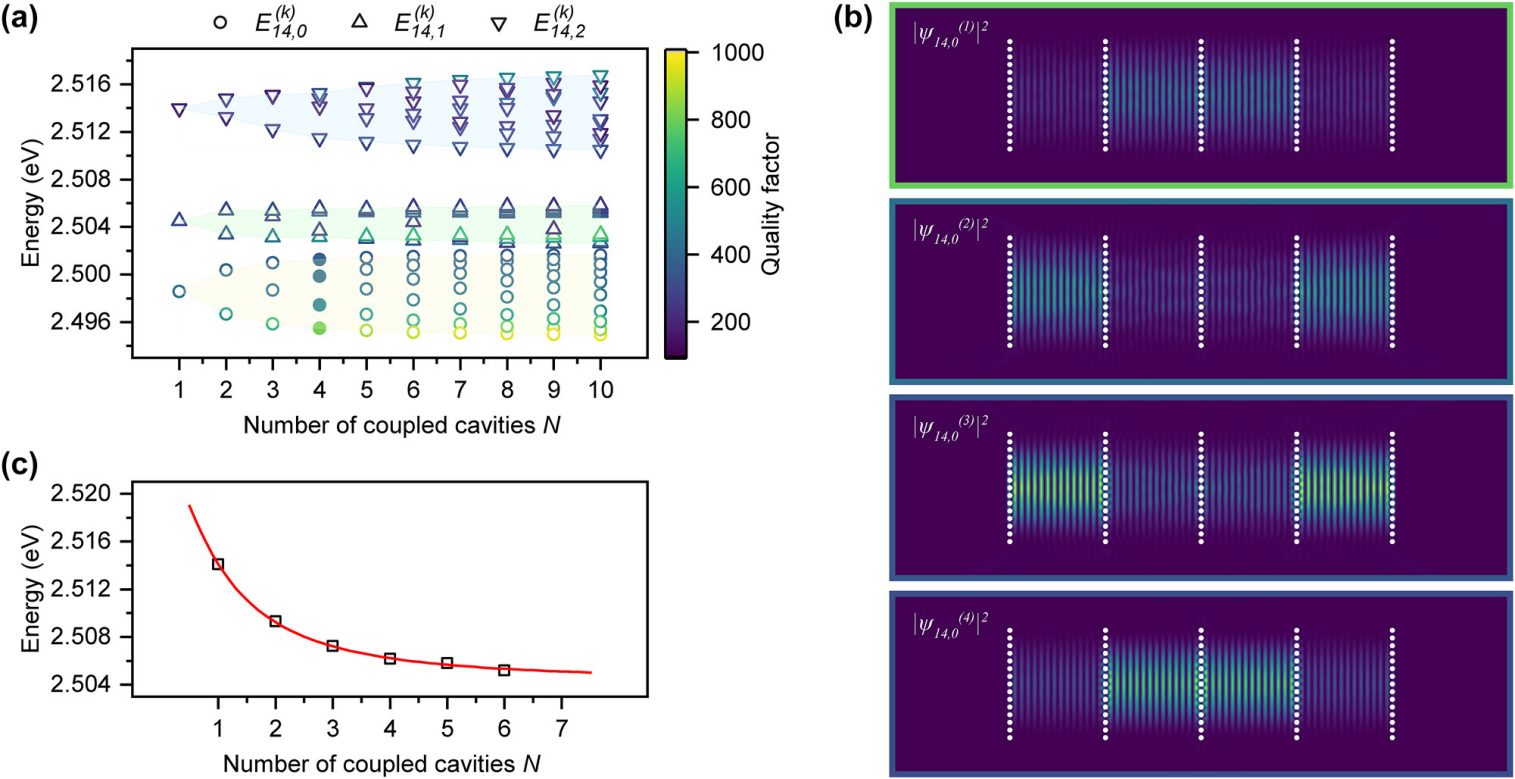
Simulated mode energies and structure. (a) Energies of the coupled modes as a function of array size *N*, extracted from transition-matrix multi-scattering simulations with complex pole finding; with color encoding the mode quality factor *Q* calculated neglecting all the out-of-plane losses. Three families of modes with different transverse orders are distinguished: 0^th^ (circles, yellow area), 1st (up triangles, green area), 2nd (down triangles, cyan area). (b) Simulated real-space intensity distributions showing the four coupled modes of lowest transversal order of a 4-coupled cavity system, from bonding to antibonding character (top to bottom) corresponding to the filled circles in a. (c) Measured condensation energy of the lowest coupled state (black squares) as a function of array size *N*, obtained from emission peaks just above threshold, compared with a tight-binding fit (red line), which gives a coupling strength of 2*J*–9.4 meV.

To rationalize these observations, we analyzed the coupled eigenmodes using a transition-matrix multi-scattering formalism combined with complex pole extraction (see Methods – Photonic Simulations) [35]. Figure 3a displays the energy as a function of array size, where the color encodes the quality factor *Q* of each resonance. In this, distinct manifolds of modes can be identified, corresponding to different transverse orders (designated by index *p*) within the same longitudinal order (index *m*). Coupling induces hybridization such that, for each transverse order, an array of *N* cavities supports *N* collective supermodes, labeled by the coupling index *k*. Notably, the energetically lowest coupled mode (*k* = 1) consistently exhibits the highest *Q* within the transverse ground state (*p* = 0). The calculated electric field intensity distributions ${\left|\psi_{m=14,p=0}^{\left(k\right)}\left(N=4\right)\right|}^{2}$ illustrate that the four supermodes of a 4-cavity array differ markedly in spatial profile (Figure 3b).

Experimentally, condensation occurs predominantly in the ground state $\psi_{m,p=0}^{\left(1\right)}\left(N\right)$ . The measured condensate emission energies, extracted from the center of the emission peak just above threshold (∼1.1 *P*_th_), red-shift systematically with increasing *N* (Figure 3c), in good agreement with a tight-binding fit (see Methods - Tight-binding analysis) [21]. This trend matches the ab-initio numerical simulations of the lowest coupled mode (Figure 3a), although the absolute energies are shifted slightly. The offset likely arises from variations in the effective refractive index due to possible thickness non-uniformities of the spin-coated polymer material and from minor fabrication discrepancies compared to the simulated cavity design. In addition, the experimentally extracted coupling strength (2*J*–9.4 meV) exceeds the simulated value of ∼4.5 meV, reflecting an overestimation of the mirror reflectivity in the numerical simulations, which neglects fabrication imperfections that eventually increase the hopping rate. Hence, the comparison confirms that the evolution of the lowest coupled state energy with cavity number ( $E_{m,p}^{\left(1\right)}\left(N\right)$ ) is captured by both the tight-binding model and the transition-matrix simulations, while higher-coupled-order states (*k* > 1) remain essentially unoccupied above threshold. Moreover, the numerically calculated cavity *Q* increases for the energetically lowest mode with the number of coupled cavities *N*. This can be qualitatively rationalized from the scaling of *Q* with the inverse of the effective cavity length in Fabry–Perot resonators [36]. Moreover, as the condensation threshold is lower for higher cavity quality factor, this rise in *Q* with *N* can indeed account for the experimentally observed threshold reduction in larger arrays.

### 2.3 Spatial and temporal coherence

**Figure 4:**
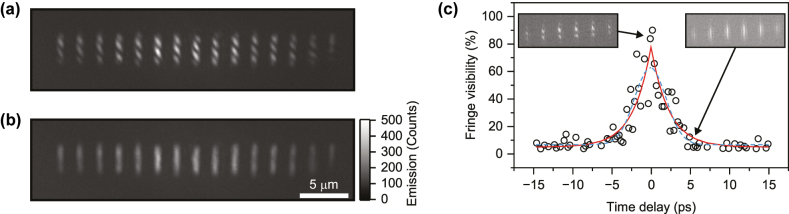
Extended coherence in a cavity array. (a) Real-space interferogram of an array of 14 coupled cavities excited by hundreds of laser pulses just above threshold (∼1.3 *P*_th_), showing interference fringes at zero time delay in a Michelson interferometer. The emission appears as bright stripes at the HCG positions because the collected far-field signal originates from out-of-plane scattering at the mirrors rather than from the intra-cavity field maxima. (b) Interferogram recorded at a delay of 4 ps, where no fringes are visible. (c) Temporal coherence of the condensate in an array of 5 coupled cavities excited with single pulses well above threshold (∼4 *P*_th_). The fringe visibility at each delay was obtained by Fourier transforming the interferogram, isolating the component at the interference fringe spatial frequency, and normalizing it to the zero-delay visibility extracted directly from the respective image from fringe minima and maxima. The red line represents the fit with exponential decays around zero, with a FWHM of 3.2 ps, whereas the blue dashed line corresponds to a Gaussian fit (FWHM of 4.7 ps). The former matches slightly better the temporal behavior of coherence, with a slightly lower residual error. The insets show single-shot interferograms at zero delay (left) and at 4 ps (right), where the respective data points are indicated by arrows.

Since extended phase coherence is a defining property of polariton condensates, we next examined the spatial and temporal coherence of the coupled arrays [21], [37], [38]. In particular, the first-order coherence of the condensate was investigated using a Michelson interferometer (see Methods – Optical Characterization), where the light in one arm was delayed and spatially inverted. Real-space interferograms recorded at zero delay (Figure 4a) and integrating the emission over hundreds of excitation pulses above the condensation threshold show clear fringes in the light scattered from the HCG mirrors. In fact, as the far-field signal mainly arises from parasitic out-of-plane scattering at the mirror positions, the resulting interferograms display bright stripes coinciding with the HCG positions. These stripes therefore mark the dominant scattering sites of the supermode rather than its intra-cavity field maxima, which are located within the cavities between the mirrors but are vertically confined through total internal reflection in the polymer. These fringes at the HCG positions confirm the presence of a single, spatially extended condensate mode across the 14-cavity array at zero delay. At longer delays, the fringe contrast decreases and vanishes above 4 ps (Figure 4b). Owing to the high saturation intensity of the organic polymer, even single-shot experiments can be performed, allowing direct observation of condensate dynamics and fluctuations without temporal averaging. Such single-shot measurements, previously demonstrated only in vertical DBR-based polariton systems [5], [39], are here realized for integrated polariton devices. For an array of 5 coupled cavities under single-shot excitation, the fringe visibility as a function of delay is plotted in Figure 4c. Single-shot interferograms at two delays are shown as insets, confirming the disappearance of fringes on the picosecond timescale. The noise present in the temporal evolution of the fringe visibility is a consequence of the low signal level in single-shot acquisition, compared to multi-shot measurements as in Figure 4a. Quantitatively, the temporal decay of the coherence follows an exponential that can be fit with a characteristic decay constant of *τ* = 2.3 ps, yielding an autocorrelation peak width with FWHM of 3.2 ps. Whereas a Gaussian decay would suggest strong inhomogeneous broadening from slow fluctuations, the observed exponential decay indicates reduced noise and a coherence time not far from being determined by the intrinsic linewidth (Fourier-limited FWHM would be *ħ*/τ ∼ 0.24 meV for a perfect Lorentzian line) of the hybridized mode [40]. Notably, because of the short polariton lifetime of hundreds of femtoseconds, the measured coherence time is mainly constrained by the excitation pulse duration of ∼5 ps that replenishes the exciton reservoir over this duration, but is also subject to dynamic instabilities [41] and other sources of noise [42]. Overall, these measurements confirm that the condensate establishes coherence over the whole array, consistent with single-mode condensation directly into a well-defined eigenmode of the coupled array and not through coupling of intermediate single-cavity condensates.

## 3 Conclusions

We have demonstrated lithographically defined arrays of on-chip microcavities based on silicon photonic fabrication technology using high-contrast gratings and organic polymer in the strong light–matter interaction regime. This integrated architecture allows us to access the cavity resonance mode through near-field techniques and provides precise control over cavity geometry and array scalability. When exciting the coupled cavities above threshold, polariton condensation occurs in modes that are delocalized across the whole array due to the coupling between the cavities, as evidenced by first-order coherence measurements. The evolution of the mode energies and thresholds with array size is rationalized by numerical simulations. These results prepare the ground for more elaborate HCG lattices that could perform more complex functionalities like ultrafast all-optical logic gates [15], [43], neuromorphic computation [44], [45], or mimic Hamiltonians that can be simulated with this room temperature polaritonic quantum fluid. As the HCG platform is suitable for the integration of a wide range of materials, and its fabrication is fully compatible with advanced photonic foundries, it provides a perspective for scalable and flexible integrated polaritonic circuits.

## 4 Methods

### 4.1 Device fabrication

The HCGs were fabricated on silicon-on-insulator (SOI) substrates comprising a 220 nm crystalline-Si device layer atop 2 µm buried oxide by means of electron-beam lithography (100 kV Raith EBPG 5200), exposing a 180–200 nm thick hydrogen silsesquioxane (HSQ) negative resist. Following development in a NaOH-based developer, the resist pattern was transferred into the top Si device layer by inductively coupled plasma reactive-ion etching with HBr, yielding cylindrical pillars ∼105 nm in diameter with a 165 nm pitch. Methyl-substituted ladder-type poly(para-phenylene) (MeLPPP; *M*_n_ = 31 500, *M*_w_ = 79 000) [46] was dissolved in toluene and spin-coated over the chip to form a conformal ∼220 nm film, as confirmed by profilometry and spectroscopic ellipsometry. Finally, a 20 nm Al_2_O_3_ encapsulation layer was deposited by electron beam evaporation to suppress photo-oxidative degradation.

### 4.2 Optical characterization

We mount the 20 × 20 mm^2^ chips, each containing hundreds of HCG cavities, on an XYZ nano-positioning stage in ambient conditions. Ultrafast excitation light pulses of 400 nm wavelength, 150 fs pulse duration, and 1 kHz repetition rate are generated from a frequency-doubled regenerative amplifier that is seeded by a mode-locked Ti:sapphire laser. We insert the pulsed light into a 25 μm core multi-mode fiber that stretches the pulse duration to several picoseconds and provides a smooth, near-Gaussian beam at its output. The incident beam, normal to the chip surface, is focused through a microscope objective (Mitutoyo Plan Apo 50×, NA = 0.7) to an approximately Gaussian spot with a FWHM diameter of ∼6 µm. The beam size can be controlled by using different objectives (i.e., Mitutoyo Plan Apo 20×), using different core size fibers (10 μm or 50 μm), and changing the collimation of the beam. Hence, the experiments were designed to ensure excitation of the whole array by controlling the pumping spot size. The emitted light, which consists of the cavity modes that are scattered by the HCG out-of-plane on top of a broad photoluminescence background, is collected via the same objective and separated from excitation via a dichroic mirror and long-pass filters. We detect the emission with a 50:50 beam splitter simultaneously with a camera and a spectrometer equipped with a CCD. For spectral measurements, we use a 1800 lines/mm grating, yielding a dispersion of ∼0.05 nm per pixel (at 490 nm). The spectrometer receives light via a multimode fiber with 10 μm, 25 μm, 50 μm, or 100 μm core diameter, resulting in detection spots on the sample of 2 μm, 5 μm, 10 μm, or 20 μm, respectively, when using the 50× objective. For the first-order coherence measurements, the signal is instead sent to a Michelson interferometer with a retroreflector in one arm path with a motorized delay stage with a resolution of Δ*x* = 100 nm, corresponding to a temporal resolution Δ*t* = 0.67 fs.

Real-space field maps were obtained with scattering-type scanning near-field optical microscopy (s-SNOM) in tapping mode with a cryo-neaSNOM from Neaspec Attocube. A platinum-coated silicon-made AFM tip (tapping frequency ∼260 kHz, amplitude ∼45 nm) was illuminated with a continuous wave (CW) laser at 488 nm (LASOS); the back-scattered light was collected in reflection and demodulated at the fourth harmonic of the tapping frequency to retrieve the near-field signal. The resulting o4 signal was recorded point-by-point while scanning.

### 4.3 Photonic simulations

For the computation of the HCG reflectivity of the cavity filled with polymer, we perform rigorous coupled wave analysis (RCWA) using a freely available software package [47]. In particular, RCWA models periodic structures by expanding the permittivity and fields into spatial Fourier harmonics. In our setup, we maintain 11 in-plane orders in *x* and *y* directions and include the complex, dispersive refractive index of silicon and the polymer material obtained from variable-angle spectroscopic ellipsometry measurements (Woollam VASE) of a test polymer layer on a silicon wafer. Gratings are solved by building per-layer Fourier-convolution matrices, finding the eigenmodes, and cascading layers with an S-matrix to enforce boundary conditions and compute diffraction efficiencies. Cavities are treated as multilayers such as Fabry–Perot or with a supercell for in-plane defects, and resonances appear as sharp features in R/T or as poles of the S-matrix.

For the numerical simulations of the resonance energies, *Q*-factors and mode fields, we used a transition-matrix multi-scattering approach with AAA pole finding, as described in more detail in [35]. In short, the arrays of HCG cavities were modelled as ensembles of cylindrical scatterers embedded in an effective background medium representing the polymer slab. Each isolated cavity supports resonances labelled with their longitudinal index *m* and transverse index *p*. When *N* identical cavities are coupled, these single-cavity modes hybridize into *N* collective eigenmodes, denoted as $\psi_{m,p}^{\left(k\right)}\left(N\right)$ with coupling index *k* = 1…*N*. The transition-matrix formalism was used to compute the multiple scattering response of the coupled cavities at complex frequencies. Subsequently the AAA algorithm for rational approximation was used to identify poles of response, corresponding to the complex resonance frequencies of the modes. In such a way, each resonance is characterized by its frequency, linewidth (from the pole’s imaginary part), and field distribution. The unnormalized simulated modal fields are presented as ${\left|\psi_{m,p}^{\left(k\right)}\left(N\right)\right|}^{2}$ .

### 4.4 Tight-binding analysis

To describe the evolution of eigenmodes with cavity number, we used a nearest-neighbor tight-binding Hamiltonian. Thus, the lowest-energy mode of each (*m*, *p*) family in an *N*-cavities array corresponds to $\psi_{m,p}^{\left(1\right)}\left(N\right)$ . Their eigenenergies are:

$$\begin{array}{ll} E_{m,p}^{\left(1\right)}\left(N\right) & =E_{m,p}^{\left(0\right)}\left(1\right)-2J_{m,p}\cos\left(\frac{k\pi }{N+1}\right)\\ & \;\;\text{for}\;k=1,\ldots ,N \end{array}$$

where $E_{m,p}^{\left(0\right)}\left(1\right)$ is the uncoupled single-cavity resonance.

## References

[1] Weisbuch C., Nishioka M., Ishikawa A., Arakawa Y. (1992). Observation of the coupled exciton-photon mode splitting in a semiconductor quantum microcavity. *Phys. Rev. Lett.*.

[2] Kasprzak J. (2006). Bose–Einstein condensation of exciton polaritons. *Nature*.

[3] Deng H., Haug H., Yamamoto Y. (2010). Exciton-polariton Bose-Einstein condensation. *Rev. Mod. Phys.*.

[4] Lidzey D. G., Bradley D. D. C., Skolnick M. S., Virgili T., Walker S., Whittaker D. M. (1998). Strong exciton–photon coupling in an organic semiconductor microcavity. *Nature*.

[5] Plumhof J. D., Stöferle T., Mai L., Scherf U., Mahrt R. F. (2014). Room-temperature Bose–Einstein condensation of cavity exciton–polaritons in a polymer. *Nat. Mater.*.

[6] Daskalakis K. S., Maier S. A., Murray R., Kéna-Cohen S. (2014). Nonlinear interactions in an organic polariton condensate. *Nat. Mater.*.

[7] Keeling J., Kéna-Cohen S. (2020). Bose–Einstein condensation of exciton-polaritons in organic microcavities. Annu. Rev. Phys. Chem..

[8] Sanvitto D., Kéna-Cohen S. (2016). The road towards polaritonic devices. *Nat. Mater.*.

[9] Kavokin A., Liew T. C. H., Schneider C., Lagoudakis P. G., Klembt S., Hoefling S. (2022). Polariton condensates for classical and quantum computing. *Nat. Rev. Phys.*.

[10] Galbiati M. (2012). Polariton condensation in photonic molecules. *Phys. Rev. Lett.*.

[11] Ohadi H. (2016). Nontrivial phase coupling in polariton multiplets. *Phys. Rev. X*.

[12] Lai C. W. (2007). Coherent zero-state and π-state in an exciton–polariton condensate array. *Nature*.

[13] Kim N. Y. (2011). Dynamical d-wave condensation of exciton–polaritons in a two-dimensional square-lattice potential. *Nat. Phys.*.

[14] Berloff N. G. (2017). Realizing the classical XY Hamiltonian in polariton simulators. *Nat. Mater.*.

[15] Zasedatelev A. V. (2019). A room-temperature organic polariton transistor. *Nat. Photonics*.

[16] Wertz E. (2010). Spontaneous formation and optical manipulation of extended polariton condensates. *Nat. Phys.*.

[17] Klembt S. (2018). Exciton-polariton topological insulator. *Nature*.

[18] Dusel M. (2020). Room temperature organic exciton–polariton condensate in a lattice. *Nat. Commun.*.

[19] Yadav R. K. (2024). Direct writing of room temperature polariton condensate lattice. *Nano Lett.*.

[20] Betzold S. (2024). Dirac cones and room temperature polariton lasing evidenced in an organic honeycomb lattice. *Adv. Sci.*.

[21] Georgakilas I. (2025). In situ tunable, room-temperature polariton condensation in individual states of a 1D topological lattice. *Sci. Adv.*.

[22] Schmutzler J. (2015). All-optical flow control of a polariton condensate using nonresonant excitation. *Phys. Rev. B*.

[23] Dreismann A. (2016). A sub-femtojoule electrical spin-switch based on optically trapped polariton condensates. *Nat. Mater.*.

[24] Gianfrate A. (2024). Reconfigurable quantum fluid molecules of bound states in the continuum. *Nat. Phys.*.

[25] Chang-Hasnain C. J., Yang W. (2012). High-contrast gratings for integrated optoelectronics. *Adv. Opt. Photonics*.

[26] Kim S., Wang Z., Brodbeck S., Schneider C., Höfling S., Deng H. (2019). Monolithic high-contrast grating based polariton laser. *ACS Photonics*.

[27] Ramezani M. (2017). Plasmon-exciton-polariton lasing. *Optica*.

[28] Väkeväinen A. I., Moilanen A. J., Nečada M., Hakala T. K., Daskalakis K. S., Törmä P. (2020). Sub-picosecond thermalization dynamics in condensation of strongly coupled lattice plasmons. *Nat. Commun.*.

[29] Castellanos G. W., Ramezani M., Murai S., Gómez Rivas J. (2023). Non-equilibrium Bose–Einstein condensation of exciton-polaritons in silicon metasurfaces. *Adv. Opt. Mater.*.

[30] Stöferle T. (2010). Ultracompact silicon/polymer laser with an absorption-insensitive nanophotonic resonator. *Nano Lett.*.

[31] Tassan P. (2025). Integrated, ultrafast all-optical polariton transistors with sub-wavelength grating microcavities. ..

[32] Abbarchi M. (2013). Macroscopic quantum self-trapping and Josephson oscillations of exciton polaritons. *Nat. Phys.*.

[33] Scafirimuto F., Urbonas D., Becker M. A., Scherf U., Mahrt R. F., Stöferle T. (2021). Tunable exciton–polariton condensation in a two-dimensional Lieb lattice at room temperature. *Commun. Phys.*.

[34] Yagafarov T. (2020). Mechanisms of blueshifts in organic polariton condensates. *Commun. Phys.*.

[35] Fischbach J. D. (2025). A framework to compute resonances arising from multiple scattering. *Adv. Theory Simul.*.

[36] Saleh B. E. A., Teich M. C. (1991). Resonator optics. *Fundamentals of Photonics*.

[37] Kasprzak J., Solnyshkov D. D., André R., Dang L. S., Malpuech G. (2008). Formation of an exciton polariton condensate: thermodynamic versus kinetic regimes. *Phys. Rev. Lett.*.

[38] Love A. P. D. (2008). Intrinsic decoherence mechanisms in the microcavity polariton condensate. *Phys. Rev. Lett.*.

[39] Estrecho E. (2018). Single-shot condensation of exciton polaritons and the hole burning effect. *Nat. Commun.*.

[40] Whittaker D. M., Eastham P. R. (2009). Coherence properties of the microcavity polariton condensate. *Europhys. Lett.*.

[41] Bobrovska N., Matuszewski M., Daskalakis K. S., Maier S. A., Kéna-Cohen S. (2018). Dynamical instability of a nonequilibrium exciton-polariton condensate. *ACS Photonics*.

[42] Putintsev A. D. (2024). Photon statistics of organic polariton condensates. *Phys. Rev. B*.

[43] Sannikov D. A. (2024). Room temperature, cascadable, all-optical polariton universal gates. *Nat. Commun.*.

[44] Ballarini D. (2020). Polaritonic neuromorphic computing outperforms linear classifiers. *Nano Lett.*.

[45] Mirek R. (2021). Neuromorphic binarized polariton networks. *Nano Lett.*.

[46] Scherf U., Bohnen A., Müllen K. (1992). Polyarylenes and poly(arylenevinylene)s, 9 the oxidized states of a (1,4-phenylene) ladder polymer. *Makromol. Chem.*.

[47] Liu V., Fan S. (2012). S4: a free electromagnetic solver for layered periodic structures. *Comput. Phys. Commun.*.

